# Implementation of multiplex PCR-based blood culture identification among hospitalized pediatric patients at a tertiary care center

**DOI:** 10.1128/spectrum.01571-26

**Published:** 2026-08-06

**Authors:** Pongsatorn Sripol, Pintip Suchartlikitwong, Napawan Punakabutra, Jiratchaya Sophonphan, Nina Ma Kang Li, Suvaporn Anugulruengkitt

**Affiliations:** 1 Department of Pediatrics, Faculty of Medicine, Chulalongkorn University 65103, Bangkok, Thailand; 2 Center of Excellence for Pediatric Infectious Diseases and Vaccines, Chulalongkorn University 26683, Bangkok, Thailand; 3 Department of Microbiology, Faculty of Medicine, Chulalongkorn University 65103, Bangkok, Thailand; 4 Research Affairs, Faculty of Medicine, Chulalongkorn University 65103, Bangkok, Thailand; 5 Medical Affairs, bioMérieux Asia Pacific Pte. Ltd., Singapore, Singapore; Icahn School of Medicine at Mount Sinai, New York, New York, USA

**Keywords:** rapid diagnostic test, pediatrics, blood culture, bacteremia

## Abstract

Conventional blood culture is the gold standard for pathogen identification for patients with sepsis, but its lengthy turnaround time—encompassing initial bottle incubation and subsequent phenotypic testing—may delay appropriate therapy. The multiplex PCR-based BIOFIRE Blood Culture Identification 2 (BCID2) panel enables earlier pathogen identification and improves patient outcomes. This study aimed to assess the impact of implementing the BCID2 panel versus conventional culture methods on clinical and microbiological outcomes in hospitalized pediatric patients. This case-control cohort study included 150 retrospective and 150 prospective hospitalized patients with positive blood cultures evaluated via parallel BCID2 and conventional culture. The antibiotic stewardship program (ASP) team provided antimicrobial recommendations based on BCID2 results, with final treatment decisions deferred to the attending physicians. Time to targeted therapy was calculated from the initial antibiotic dose—or blood culture collection if the patient was already on antibiotics—to the start of targeted antimicrobial therapy. The median age in the BCID2 group was significantly higher than the pre-BCID2 group, 2.8 vs 1.7 years, respectively (*P* = 0.032). While 18 pathogens among the 150 BCID2 cases (12%) were not identified due to being off-panel targets, BCID2 results prompted antimicrobial modification in 28.8% of the remaining cases (38/132). Median time to targeted therapy was 17.0 hours (IQR 9.6–34.2) with BCID2 group versus 24.2 hours (IQR 16.3–52.2) with conventional culture (*P* = 0.014). On multivariable logistic regression analysis, the BCID2 intervention remained an independent and significant predictor of achieving rapid antimicrobial optimization (aOR 2.68; 95% CI 1.17–6.14; *P* = 0.019). BCID2 implementation paired with an active ASP optimizes antimicrobial use in pediatric bacteremia through accelerated targeted therapy. However, a negative BCID2 result requires nuanced clinical interpretation due to the possible presence of off-panel pathogens or rare genotype–phenotype discrepancies.

**IMPORTANCE:**

Bloodstream infection (BSI) remains a leading cause of pediatric morbidity and mortality worldwide. While conventional blood culture is the gold standard, its lengthy turnaround time often delays life-saving therapy. Rapid diagnostic testing (RDT), such as the BIOFIRE Blood Culture Identification 2 (BCID2) panel, is now recommended alongside antimicrobial stewardship programs (ASPs) to optimize clinical management. Our study demonstrates that implementing multiplex PCR-based RDT significantly improves patient outcomes by accelerating the time to targeted therapy. Furthermore, the integration of RDT into an ASP was associated with a significant reduction in both 30-day mortality and hospital length of stay. In conclusion, RDT-guided stewardship serves as both a vital diagnostic tool and a clinical engine that transforms the management of pediatric BSI.

## INTRODUCTION

Bloodstream infection (BSI) is a major problem that causes significant morbidity and mortality in children worldwide (1). The World Health Organization (WHO) reported in 2017 that nearly half of all global sepsis cases occurred among children, with an estimated 20 million cases and 2 million deaths worldwide, contributing to longer hospital stays and higher hospital costs (2, 3). Conventional blood culture is the gold-standard laboratory tool for identifying bacteria causing BSI. In standard laboratory workflows, once an automated blood culture system signals positive, subculture on solid agar media is required for conventional organism identification and antimicrobial susceptibility testing. This traditional process generally takes 48–72 hours from the time of bottle positivity, leading to delays in initiating appropriate antibiotic therapy and overall clinical management. Nowadays, many diagnostic tools are available to identify these organisms, allowing for earlier diagnosis and more effective use. Rapid diagnostic testing (RDT), such as multiplex PCR panels, is recommended to be used in conjunction with conventional blood cultures. The Infectious Disease Society of America also recommends adding RDT to an effective antimicrobial stewardship program (ASP) (4). One such RDT is the BIOFIRE Blood Culture Identification 2 (BCID2) panel, which can identify 43 targets associated with bloodstream infections (5). Studies have shown that implementing BCID2 in clinical practices can help clinicians optimize antibiotic use, reducing exposure to inappropriate antibiotics, lowering the risk of multidrug-resistant bacteria, and improving clinical outcomes (6). However, it is important to consider that the epidemiology of bacterial infections may differ geographically. In Thailand, there is a rising prevalence of critically drug-resistant gram-negative bacteria. National surveillance data show that carbapenem resistance rates are increasing in recent few years (7). Data from the National Antimicrobial Resistance Surveillance Center, Thailand, indicate that gram-negative bacteria are the leading cause of BSI nationwide. Among pediatric BSI cohorts, the top three isolated pathogens are *Escherichia coli* (24.9%), *Acinetobacter baumannii* (23.7%), and *Klebsiella pneumoniae* (20.7%). Crucially, the prevalence of extended-spectrum beta-lactamase production among *E. coli* isolates stands at 35.2%, while carbapenem-resistant *Enterobacterales* account for 15.9% of cases (7). Therefore, further study is needed to specifically evaluate the clinical impact of BCID2 implementation compared to conventional blood culture methods.

## MATERIALS AND METHODS

### Setting

This single-center, quasi-experimental pre–post interventional study evaluated the clinical impact of implementing BCID2 in routine clinical practice. The study was conducted at King Chulalongkorn Memorial Hospital (KCMH), a tertiary-care teaching hospital in Bangkok, Thailand, which includes a 200-bed pediatric service with one pediatric intensive care unit (PICU). At our institution, empirical antibiotic therapy for suspected pediatric sepsis is stratified by clinical onset and severity. For community-acquired sepsis, a third-generation cephalosporin is typically initiated. For hospital-acquired sepsis, empirical coverage escalates to piperacillin/tazobactam or a carbapenem. In cases presenting with severe sepsis, septic shock, or known prior colonization with multidrug-resistant pathogens, a carbapenem is prescribed, occasionally combined with an aminoglycoside. Vancomycin is added empirically if a central line-associated bloodstream infection or a resistant gram-positive pathogen is suspected. The study was approved by the Institutional Review Board of the Faculty of Medicine, Chulalongkorn University (IRB no. 0501/67).

### Population

The study population included hospitalized pediatric patients aged <18 years admitted to general pediatric wards or the PICU with positive blood culture. Exclusion criteria comprised patients admitted to the nursery, special care unit, or neonatal intensive care unit (NICU). These wards were excluded because active antimicrobial stewardship rounds were restricted to pediatric units at our institution. Additionally, incomplete medical records regarding primary outcomes, specifically missing data on antimicrobial utilization, were excluded. Two cohorts were included: (1) the pre-BCID2 implementation group, consisting of 150 hospitalized children with positive blood cultures collected between 2023 and 2024 and (2) the BCID2 implementation group, consisting of 150 hospitalized children with positive blood cultures consecutively enrolled between October 2024 and October 2025. Duplicate blood culture isolates from the same patient were excluded. During the BCID2 implementation phase, conventional blood culture and BIOFIRE BCID2 panel (bioMérieux, Marcy l’Etoile, France) were performed in parallel. The BCID2 test results were communicated to the primary physician.

### Conventional blood culture methods

BD BACTEC Peds Plus/F culture vials were incubated in the BD BACTEC blood culture system (Becton, Dickinson and Company, Franklin Lakes, NJ, USA). When bottles flagged positive, aliquots were prepared for microscopy and Gram staining, and results were reported to the primary treating physician as critical values within 30 minutes. Positive cultures were subsequently subcultured onto blood agar, chocolate, and MacConkey agar plates and incubated at 35°C in ambient air with 5% CO_2_ for 16–20 hours to allow organism isolation prior to identification and antimicrobial susceptibility testing. Bacteria and yeasts were identified using matrix-assisted laser desorption/ionization time-of-flight mass spectrometry (MALDI-TOF MS; VITEK MS, bioMérieux, Marcy l’Étoile, France). Phenotypic antimicrobial susceptibility testing was performed using the VITEK 2XL automated system (bioMérieux, Marcy-l'Étoile, France) with AST-N416 cards for Enterobacterales and AST-P592 cards for *Staphylococcus*. Susceptibility testing for *Enterococcus* was performed using ETEST strips (bioMérieux, Marcy-l'Étoile, France), while *Salmonella, Pseudomonas aeruginosa*, and *Acinetobacter* were tested using the disk diffusion method. Antimicrobial susceptibility results were interpreted according to the Clinical and Laboratory Standards Institute (CLSI) M100 guidelines (8).

### BIOFIRE BCID2 panel testing

Testing was performed according to the manufacturer’s instructions. Briefly, a 200 µL aliquot from a positive blood culture bottle was mixed with dilution buffer and loaded into an FA-PP cartridge. Nucleic acid extraction, amplification, detection, and analysis were fully automated within the cartridge, with a total run time of approximately 1 hour. The BCID2 test was performed on the BIOFIRE FILMARRAY 2.0 system (bioMérieux, Salt Lake City, UT, USA) using software version 3.3.8.0 and the BCID2 panel version 1.0. Results were reported qualitatively as “detected” or “not detected.” In accordance with the manufacturer’s instructions for use, BCID2 results were interpreted in conjunction with the initial Gram stain results. The BIOFIRE BCID2 panel targets 26 bacterial pathogens, 7 yeasts, and 10 antimicrobial resistance genes, including extended-spectrum β-lactamases, carbapenemases, and determinants of vancomycin, methicillin, and colistin resistance, with an overall pooled sensitivity of 99.0% (95% confidence interval [CI]: 97.8%–99.6%) and an overall specificity of 99.8% (95% CI: 99.7%–99.9%) for target identification (5).

### Study protocol

#### Pre-BCID2 implementation period

A retrospective data review of 150 hospitalized eligible patients with positive blood culture during 2023–2024 (before BCID2 implementation) was performed. Bacterial identification was performed using the conventional blood culture method. Investigators reviewed demographic data, pathogens, antimicrobial susceptibility test results, antimicrobial adjustment, and time to pathogen identification/time to targeted antimicrobial therapy. Patients with insufficient clinical data in the pre-BCID2 implementation period were excluded from the study.

#### BCID2 implementation period

A total of 150 hospitalized children with positive blood cultures were prospectively enrolled, with conventional blood cultures and BCID2 test performed in parallel. At the study hospital, ASP provides clinical practice guidelines training for pediatric residents and conducts regular antimicrobial stewardship ward rounds. For this study, a BCID2 results interpretation guide was developed, and the ASP team provided real-time recommendations based on the BCID2 results during working hours and within 8 hours during night shifts. Subsequent antimicrobial therapy was adjusted based on the decision of the attending physicians. Therapy modification was monitored by the ASP team within 24 hours following each recommendation. In cases where discrepancies occurred between the BCID2 panel results and final conventional cultures, a standardized resolution protocol was deployed. The microbiology laboratory verified the initial Gram stain, evaluated subculture agar plates for potential polymicrobial or fastidious organisms, and repeated testing if a technical artifact was suspected. Clinically, whenever a genotypic–phenotypic mismatch occurred, antibiotic management was guided strictly by the initial Gram stain morphology and maintained on broad-spectrum empiric regimens until final phenotypic identification and susceptibility testing were available.

The study was initially designed as a matched case-control study, with plans to employ frequency matching based on age, immune status, and ward type (PICU vs non-PICU). However, strict frequency matching could not be fully achieved due to data limitations in the pre-BCID2 cohort and a shift in patient demographics during the BCID2 period. Specifically, our institution experienced an increased volume of complex, critically ill pediatric referrals. To account for these baseline imbalances, age, immune status, and ICU admission were further controlled for using multivariable logistic regression in the final analysis.

### Data analysis

Clinical outcomes were compared between the pre-BCID implementation period and BCID2 implementation periods with regard to (i) time to targeted antimicrobial therapy, (ii) antimicrobial modification according to appropriate antibiotic switching based on organism susceptibility, and (iii) other outcomes including 30-day mortality, length of hospital stays, and direct hospital costs. Microbiological outcomes focused on the concordance between BCID2 and conventional cultures, with a detailed analysis of result discordances and discrepancies.

### Outcomes definition

#### Time to targeted antimicrobial therapy

Defined as the time from the initial antibiotic dose—or blood culture collection if the patient was already on antibiotics—to the first dose of targeted antimicrobial therapy or antimicrobial discontinuation for contaminants (9).

#### Targeted antimicrobial therapy

Defined as an antimicrobial therapy covering the causative pathogen(s) according to Moehring et al. (10).

#### Antimicrobial modification

The categories of change were as follows: (i) Escalation: changing therapy from a narrow-spectrum antibiotic to a broader-spectrum antibiotic, (ii) De-escalation: changing therapy from a broader-spectrum antibiotic to a more narrow-spectrum antibiotic according to the spectrum table (10), (iii) Discontinuation: ceasing antimicrobial therapy, and (iv) Others, including adding vancomycin and adding antifungal agents. Cases in which the BCID2 panel yielded a “no target detected” result were excluded from the antimicrobial modification analyses. Because a negative BCID2 from a positive bottle indicates the potential presence of an off-panel organism, antibiotic management for these patients remained unchanged and was guided strictly by the initial Gram stain and final phenotypic culture results.

#### 30-day mortality rate

The 30-day period begins on the date pathogens were identified by conventional culture. Mortality was tracked for all patients during this period regardless of whether the patient was hospitalized or had been discharged.

#### Length of stay

The date from blood culture positivity to discharge date.

#### Direct hospital cost

Individual patient costs were retrieved from electronic medical records. This includes direct medical costs (e.g., medications, diagnostic tests, medical supplies, and physician fees) and direct non-medical costs incurred by the facility (e.g., room charges, administrative costs related to patient care).

#### MDR pathogen

Defined as a pathogen that was characterized as resistant to more than one antimicrobial agent or resistant to three or more antimicrobial classes based on *in vitro* antimicrobial susceptibility test results (11).

#### Contaminated blood cultures

Defined as growth of commensal organisms such as coagulase-negative staphylococci from a single blood culture set when ≥2 blood culture sets were collected, except among participants suspected to have true bacteremia associated with central venous catheters or devices (12).

### Statistical analysis

Sample size was calculated by using “G*Power program” for statistical power analyses and calculating sample size with the Wilcoxon–Mann–Whitney test (two independent groups) (13). The numbers we used in the program were referred to Messacar et al. (14); the mean time to targeted antibiotic prescription decreased from 60.2 hours in the control group to 26.7 hours in the intervention group. Data were compared by replacing pre-BCID2 and BCID2 implementation groups with mean and standard deviation from these two groups. The standard deviation was calculated by formula $\sqrt{N}x\;\left(upper\;limit-lower\;limit\right)$, with the calculated effect size being 0.358, alpha error being 0.05, power being 0.8, and allocation ratio being 1:1. Accounting for an anticipated 10% incomplete data or drop-out rate, a final sample size of 150 subjects per group was chosen. Statistical comparisons were performed between study groups with the Mann–Whitney test for continuous variables. Categorical variables were compared using the Chi-square test. Continuous variables were compared using multivariable logistic regression with 95% CI. Multivariable analyses included variables with *P* < 0.2 from univariable analysis. Statistical analysis was performed using Stata software, version 19.5 (Stata Corp LLC, College Station, TX) (15). A *P*-value < 0.05 was considered statistically significant.

## RESULTS

### Baseline characteristics

A total of 300 patients were analyzed, with 150 included in each period. The median age in the BCID2 group was significantly higher than the pre-BCID2 group, 2.8 vs 1.7 years, respectively (*P* = 0.032). Underlying diseases are shown in Table 1.

**TABLE 1:** Demographic of participants between pre- and post-implementation group^*a*^

Baseline characteristics	Pre-BCID2 implementation	BCID2 implementation	*P*-value^*b*^
Age, median (IQR) in months	1.7 (0.4–5.5)	2.8 (0.8–9.6)	0.032^†^
Gender (*n*, %)			0.106
Male	69 (46%)	83 (55.3%)	
Female	81 (54%)	67 (44.7%)	
ICU admission (*n*, %)			<0.001
ICU	10 (6.7%)	54 (36%)	
Non-ICU	140 (93.3%)	96 (64%)	
Host status (*n*, %)			0.537
Immunocompetent	99 (66%)	104 (69.3%)	
Immunocompromised	51 (34%)	46 (30.7%)	
Underlying disease (*n*, %)			
Hematology-oncology	40 (26.7%)	34 (22.6%)	0.422
Heart disease	35 (23.3%)	27 (18%)	0.254
Gastrointestinal disease	32 (21.3%)	25 (16.7%)	0.303
Pulmonary disease	9 (6%)	21 (14%)	0.021
Neurologic disease	5 (3.3%)	10 (6.7%)	0.185
Kidney disease	4 (2.7%)	6 (4%)	0.520
Others (endocrine/skin, etc.)	19 (12.7%)	17 (11.3%)	0.722
No underlying disease	6 (4%)	10 (6.7%)	0.304
Contaminated blood cultures, *n* (%)	35 (23.3%)	40 (26.7%)	0.505

^*a*^ IQR; interquartile range.

^*b*^ *P*-value calculated by Chi-square test or Fisher’s exact test as appropriate, except where indicated by † by Mann–Whitney test.

The proportion of patients admitted to the ICU was significantly higher in the BCID2 group as compared to the pre-BCID2 group (36.0% vs 6.7%, *P* < 0.001) as our institution experienced an increased volume of complex, critically ill pediatric referrals during the study period. There were no significant differences in rates of contaminated blood cultures between the pre-BCID2 and BCID2 groups (23.3% vs 26.7%, *P* = 0.505).

### Clinical outcomes

BCID2 implementation significantly improved the time to targeted antimicrobial therapy (Table 2). The median duration of targeted antimicrobial therapy decreased significantly from 24.2 hours (IQR 16.3–52.2) in the pre-BCID2 group to 17.0 hours (IQR 9.6–34.2) in the BCID2 group (*P* = 0.014). The proportion of patients receiving targeted antimicrobial therapy within 24 hours increased from 4.7% in the pre-BCID2 group to 20.7% in the BCID2 group (*P* < 0.001). Regarding the antimicrobial modifications, after excluding 18 negative BCID2 results, BCID2 implementation guided modifications in 28.8% of cases (38/132). The observed antimicrobial modifications occurred predominantly among patients with gram-negative bacteremia (48.3%), which constitutes the primary burden of bloodstream infections in our clinical setting. This modification comprised de-escalation (12/38, 31.6%), escalation (13/38, 34.2%), adding vancomycin (7/38, 18.4%), adding antifungal agents (5/38, 13.2%), and discontinuation (1/38, 2.6%).

**TABLE 2:** Clinical outcomes between pre- and post-implementation group^*a*^

Outcomes	Pre-BCID2 implementation (*n* = 150)	BCID2 implementation (*n* = 150)	*P*-value^*b*^
Empirical antibiotic before Gram stain identification, *n* (%)	35 (23.3%)	38 (25.3%)	0.686
Duration to targeted antimicrobial, hours, median (IQR)	24.2 (16.3–52.2)	17.0 (9.6-34.1)	0.014^†^
Targeted antimicrobial changing after conventional culture or BCID2 results, *n* (%)			
Within 6 hours	4 (2.7)	26 (17.3)	<0.001
Within 24 hours	7 (4.7)	31 (20.7)	<0.001
More than 24 hours	13 (8.7)	36 (24.0)	<0.001

^*a*^ IQR; interquartile range.

^*b*^ *P*-value calculated by Chi-square test or Fisher’s exact test as appropriate, except where indicated by †, which was calculated by Mann–Whitney test.

Factors associated with targeted antimicrobial therapy are shown in Table 3. In the multivariable logistic regression analysis, age and ICU status were included to adjust for confounders. The analysis demonstrated that the BCID2 implementation period was an independent predictor associated with an increased likelihood of targeted therapy. Targeted antimicrobial therapy was significantly more prevalent in the BCID2 group than in the pre-BCID2 group (aOR 2.68; 95% CI 1.17–6.14; *P* = 0.019). Other potential confounding variables—specifically age, sex, and ICU admission status—were not significantly associated with targeted antimicrobial therapy (*P* > 0.05).

**TABLE 3:** Factors associated with targeted antimicrobial therapy^*a*^

	Univariable		Multivariable	
	OR (95% CI)	*P*-value	Adjusted OR (95% CI)	*P*-value
Period				
– Pre-BCID2 implementation	1	Ref		
– BCID2 implementation	2.67 (1.23–5.79)	0.013	2.68 (1.17–6.14)	0.019
Age	1.01 (0.95–1.09)	0.623	0.98 (0.91–1.07)	0.720
Gender				
– Female	1	Ref		
– Male	1.92 (0.91–4.03)	0.086	1.81 (0.85–3.85)	0.125
Intensive care unit admission				
– ICU	1	Ref		
– Non-ICU	1.38 (0.61–3.12)	0.439	0.88 (0.37–2.12)	0.782
Host status				
– Immunocompetent	1	Ref		
– Immunocompromised	1.00 (0.47–2.14)	0.998		
Underlying disease				
– Hemato-Oncology	1.32 (0.58–2.98)	0.505		
– Heart disease	1.84 (0.84–3.99)	0.126		
– Gastrointestinal disease	1.18 (0.53–2.67)	0.683		
– Pulmonary disease	0.26 (0.04–2.04)	0.204		
– Neurologic disease	0.35 (0.05–2.72)	0.318		
– Kidney disease	1.22 (0.13–9.41)	0.916		

^*a*^ OR; odds ratio, CI; confidence interval.

Subgroup analysis revealed the positive impact of BCID2 implementation was most pronounced in specific populations (Table 4). A statistically significant increase in targeted therapy was observed in children aged >1 year (OR 2.77; 95% CI 1.05–7.35, *P* = 0.020) and patients in non-ICU settings (OR 2.91; 95% CI 1.23–6.90, *P* = 0.015). Moreover, immunocompromised patients derived the greatest benefit from the BCID2 test, with OR of 13.89 (95% CI 1.70–113.39, *P* = 0.014) for receiving targeted therapy in the BCID2 implementation period.

**TABLE 4:** Subgroup analysis of the association between BCID2 implementation and targeted antimicrobial therapy

	*n*	*n* (%) targeted therapy	OR (95% CI)	*P*-value
Age <1 year				
Pre-BCID2	54	4 (7.4)	1	Ref
BCID2	41	7 (17.1)	2.57 (0.70-9.47)	0.155
Age > 1 year				
Pre-BCID2	96	6 (6.3)	1	Ref
BCID2	109	17 (5.6)	2.77 (1.05–7.35)	0.020
ICU status				
Pre-BCID2	10	1 (10)	1	Ref
BCID2	54	8 (14.8)	1.56 (14.1–17.4)	0.690
Non-ICU status				
Pre-BCID2	140	9 (6.4)	1	Ref
BCID2	96	16 (16.7)	2.91 (1.23–6.90)	0.015
Immunocompetent				
Pre-BCID2	99	9 (9.1)	1	Ref
BCID2	104	14 (14.5)	1.55 (0.64–3.76)	0.329
Immunocompromised				
Pre-BCID2	51	1 (2.0)	1	Ref
BCID2	46	10 (21.7)	13.89 (1.70–113.39)	0.014

There was a statistically significant reduction in the overall 30-day mortality rate, decreasing from 8.0% in the pre-BCID2 group to 2.7% in the BCID2 group (*P* = 0.04). The median (IQR) length of stay was significantly shorter in the BCID2 group compared to the pre-BCID2 group (36 [16–68]) vs 46 [22–76] days; *P* = 0.006). From an economic perspective, BCID2 implementation resulted in a substantial reduction in overall direct hospital costs, decreasing from 18,214 USD (IQR 5,628–33,873) per patient in the pre-BCID2 group to 12,360 USD (IQR 3,279–26,965) per patient in the BCID2 group (*P* = 0.017).

### Detection of organisms and antimicrobial resistance genes using the BCID2 panel in positive blood cultures

Among 150 blood cultures included in the BCID2 implementation period, conventional culture yielded a total of 170 organisms. Polymicrobial growth was observed in 16 cultures (10.7%), yielding 36 organisms in total (ranging from 2 to 4 organisms per culture). Out of the 170 total isolates. Among these, 144 organisms (84.7%) were on-panel targets, while the remaining 26 isolates (15.3%) were off-panel organisms (such as *Aeromonas* spp. and *Burkholderia cepacia* group). Overall, the BCID2 panel achieved an isolate detection rate of 81.2% (138/170) across all cultured pathogens, accounting for both on- and off-panel targets: the BCID2 test demonstrated an on-panel concordance rate of 95.8% (138/144). At the blood culture level, the BCID2 test correctly identified pathogens in 123 of 150 blood cultures, including 82 (59.4%) gram-positive bacteria, 47 (34.1%) gram-negative bacteria, and 9 (6.5%) Candida species. Sixty-one antimicrobial resistance genes were detected in 59 blood cultures, including *mecA/C* (*n* = 44), *bla*_CTX-M_ (*n* = 8), *bla*_NDM_ (*n* = 6), *bla*_OXA-48-like_ (*n* = 2), and *vanA/B* (*n* = 1). Additional details are provided in Supplemental material.

## DISCUSSION

This quasi-experimental pre-/post-implementation study demonstrated that BCID2 implementation in the pediatrics department at a tertiary care center significantly reduced time to targeted antimicrobial therapy and guided antimicrobial modification in approximately one-third of cases. Additionally, BCID2 implementation was also associated with improved clinical outcomes, including reduced 30-day mortality, length of stay, and hospital costs.

This study highlights that BIOFIRE BCID2 panel significantly improved antibiotic treatment in clinical practice. Time to targeted antimicrobial treatment decreased significantly from 24.2 hours in the pre-BCID2 group to 17.0 hours in the BCID2 group, representing a 30% reduction. This finding aligns with previous studies (14, 16) reporting a significant reduction in the time to optimal therapy. Regarding BCID2-guided antimicrobial modifications, our study reported antimicrobial modifications in 28.8% of cases. In comparison, Sparks et al. (17) reported antibiotic modifications in 43.1% of cases. The differences across studies may be attributed to the rate of appropriate initial empirical antibiotics prescribed to patients; patients required neither escalation nor de-escalation of therapy following pathogen identification and susceptibility testing. When comparing the proportion of targeted antimicrobial modifications between the two groups, treatment modification occurred less frequently in the pre-BCID group. This lower rate of early optimization can be attributed to the lack of a real-time ASP feedback loop. In contrast, combining the rapid results of the BCID2 panel with active ASP feedback empowered clinicians to confidently make the necessary antimicrobial adjustments significantly faster than those in the pre-BCID2 group. Overall, the BCID2 panel facilitated rapid antimicrobial optimization upon positive blood culture notification, circumventing the standard incubation periods required for conventional culture-based workflows. Integrating this molecular diagnostic tool with active ASP provides the critical operational engine to translate rapid data into improved clinical outcomes. Looking forward, diagnostic workflows could be further optimized by validating protocols that utilize short-incubation subcultures (e.g., identifying pathogens from scant growth on agar plates). When paired with rapid phenotypic testing, such strategies could significantly abbreviate laboratory turnaround times and complement molecular assays.

Due to unbalanced demographic data on age and ICU status among the pre-BCID and BCID2 groups, multivariate logistic regression was used to identify factors influencing antimicrobial modification. After adjusting for demographic variables (age and ICU status), it was concluded that these factors did not independently influence targeted antimicrobial therapy. Subgroup analysis also revealed that immunocompromised patients received greater benefit from targeted therapy from BCID2-guided therapy compared to those from conventional blood culture, with approximately 14-fold improvement. As supported by previous literature (12, 14, 16, 17), BCID2 implementation significantly decreases the time to targeted antibiotic therapy. By facilitating more rational and targeted treatment decisions, the BCID2-driven ASP team contributed to a reduction in overall mortality rates and a marked improvement in overall patient clinical outcomes. This study also demonstrated a significant reduction in 30-day mortality (from 8.0% to 2.7%), consistent with data showing decreased in-hospital mortality (18). While some studies reported no mortality benefits (19, 20), those discrepancies may likely stem from confounding medical comorbidities or delayed targeted therapy for off-panel pathogens requiring conventional blood culture identification. Our finding that the length of stay was significantly shorter in the BCID2 group aligns with recent meta-analysis data demonstrating that RDT combined with an ASP significantly reduces hospital stay compared to conventional blood culture alone (OR, 0.91; 95% CI 0.84–0.98) (18). Regarding hospital economics, we observed a 32% reduction in total hospital costs per patient when comparing the pre-BCID2 group to the BCID group. While implementing the BCID2 panel incurs higher up-front laboratory expenditure, these initial costs are effectively offset by shorter hospital stays and improved clinical outcomes.

In terms of the impact of implementation of the BCID2 panel versus conventional culture methods on microbiological outcomes among hospitalized pediatric patients, the BCID2 panel demonstrated good overall concordance with conventional culture for organism identification, correctly identifying isolates. The remaining were primarily due to off-panel pathogens, which are not represented in the panel, rather than analytical misidentification. In contrast, prior studies have reported that missed on-panel isolates occur predominantly in polymicrobial cultures (19, 20). This emphasizes that rapid molecular tests must supplement, rather than replace, conventional culture methods. Clinicians must remain cautious when a BCID2 returns “no target detected” from a positive blood culture bottle, as this can stem from the presence of off-panel pathogens or rare genotype–phenotype discrepancies.

Our findings represent a comprehensive evaluation of integrating the BCID2 panel with an active ASP in a tertiary pediatric care setting. The use of the BCID2 allowed for the detection of a broader range of antimicrobial resistance markers, which is critical in managing the high prevalence of multidrug-resistant organisms observed in our pediatric patient population.

Nevertheless, the study is subject to some limitations. Hospital costs were extracted from electronic medical records and may be confounded by patient comorbidities or other clinical factors unrelated to the bacteremia episodes. However, it is notable that overall costs remained lower in the BCID2 group despite a higher baseline proportion of ICU admissions in the BCID2 group. Additionally, as the study was conducted in a single tertiary care hospital with a high prevalence of MDR organisms, these findings may not be fully generalizable to healthcare settings with different epidemiological profiles.

In conclusion, integrating BCID2 into a pediatric ASP facilitates earlier targeted antimicrobial therapy and reduces broad-spectrum antibiotic exposure. This approach not only enhances patient safety through reduced mortality but also lowers healthcare costs. The high-concordance results between BCID2 and conventional blood culture results support the use of BCID2 as a diagnostic tool in clinical practice.

## Data Availability

The data that support the findings of this study are available from the corresponding author upon reasonable request.

## SUPPLEMENTAL MATERIAL

Supplemental text and Table S1 spectrum.01571-26-s0001.docx Results of organism and antimicrobial resistance gene detection by BCID2 compared with conventional culture.

ASM does not own the copyrights to Supplemental Material that may be linked to, or accessed through, an article. The authors have granted ASM a non-exclusive, world-wide license to publish the Supplemental Material files. Please contact the corresponding author directly for reuse.
