# Supplementary material for: Implementation of multiplex PCR-based blood culture identification among hospitalized pediatric patients at a tertiary care center
Source: Microbiol Spectr. 2026 Aug 6;14(9):e01571-26. doi: 10.1128/spectrum.01571-26 (PMC13532349; doi:10.1128/spectrum.01571-26)
Supplement: Supplementary file 1 [file spectrum.01571-26-s0001.docx]

**Supplementary Table S1**

**Results of organism and antimicrobial resistance gene detection by BCID2 compared with conventional culture (n=150).**

| **Blood culture no.** | **Conventional culture results** | **RDT assay results** | **Resistance genes** |
| --- | --- | --- | --- |
| 1 | *Staphylococcus epidermidis* | *Staphylococcus* spp.  *Staphylococcus epidermidis* | *mecA/C* |
| 2 | *Staphylococcus capitis* | *Staphylococcus* spp. | N/A |
| 3 | *Candida parapsilosis* complex | *Candida parapsilosis,*  *Candida tropicalis* | N/A |
| 4 | *Stenotrophomonas maltophilia* | *Stenotrophomonas maltophilia* | N/A |
| 5 | *Klebsiella pneumoniae* | *Enterobacterales*  *Klebsiella pneumoniae* group | Not detected |
| 6 | *Candida tropicalis* | *Candida tropicalis* | N/A |
| 7 | *Staphylococcus aureus* | *Staphylococcus* spp.  *Staphylococcus aureus* | Not detected |
| 8 | *Staphylococcus capitis* | *Staphylococcus* spp.  *Staphylococcus epidermidis* | *mecA/C* |
| 9 | *Staphylococcus epidermidis* | *Staphylococcus* spp.  *Staphylococcus epidermidis* | *mecA/C* |
| 10 | *Candida parapsilosis* complex | *Candida parapsilosis* | N/A |
| 11 | *Salmonella* spp. | *Enterobacterales*  *Salmonella* spp. | Not detected |
| 12 | *Staphylococcus haemolyticus* | *Staphylococcus* spp.  *Staphylococcus epidermidis* | *mecA/C* |
| 13 | *Staphylococcus haemolyticus,*  *Acinetobacter baumannii* complex | *Staphylococcus* spp.,  *Acinetobacter calcoaceticus-baumannii* complex | NDM |
| 14 | *Staphylococcus epidermidis* | *Staphylococcus* spp.  *Staphylococcus epidermidis* | *mecA/C* |
| 15 | *Escherichia coli* | *Enterobacterales*  *Escherichia coli* | CTX-M |
| 16 | *Moraxella osloensis* | *Candida parapsilosis* | N/A |
| 17 | *Staphylococcus epidermidis* | *Staphylococcus* spp.  *Staphylococcus epidermidis* | *mecA/C* |
| 18 | *Staphylococcus epidermidis,*  *Staphylococcus hominis,*  *Staphylococcus lugdunensis* | *Staphylococcus* spp.  *Staphylococcus epidermidis*,  *Staphylococcus lugdunensis* | *mecA/C* |
| 19 | *Staphylococcus epidermidis* | *Staphylococcus* spp.  *Staphylococcus epidermidis* | *mecA/C* |
| 20 | *Staphylococcus haemolyticus* | *Staphylococcus* spp.  *Staphylococcus epidermidis* | *mecA/C* |
| 21 | *Streptococcus pneumoniae* | *Streptococcus* spp.  *Streptococcus pneumoniae* | N/A |
| 22 | *Acinetobacter baumannii* complex,  *Pseudomonas aeruginosa* | *Acinetobacter calcoaceticus-baumannii* complex,  *Pseudomonas aeruginosa* | NDM |
| 23 | *Candida glabrata* | *Candida glabrata* | N/A |
| 24 | *Escherichia coli* | *Enterobacterales*  *Escherichia coli* | CTX-M |
| 25 | *Staphylococcus epidermidis* | *Staphylococcus* spp.  *Staphylococcus epidermidis* | *mecA/C* |
| 26 | *Staphylococcus epidermidis,*  *Rothia mucilaginosa* | *Staphylococcus* spp.  *Staphylococcus epidermidis* | *mecA/C* |
| 27 | *Stenotrophomonas maltophilia* | *Stenotrophomonas maltophilia* | N/A |
| 28 | *Enterococcus faecium* | *Enterococcus faecium* | Not detected |
| 29 | *Acinetobacter baumannii* complex | *Acinetobacter calcoaceticus-baumannii* complex | NDM |
| 30 | *Staphylococcus epidermidis* | *Staphylococcus* spp.  *Staphylococcus epidermidis* | *mecA/C* |
| 31 | *Staphylococcus epidermidis,*  *Staphylococcus hominis* | *Staphylococcus* spp.  *Staphylococcus epidermidis* | *mecA/C* |
| 32 | *Stenotrophomonas maltophilia* | *Stenotrophomonas maltophilia* | N/A |
| 33 | *Staphylococcus warneri* | *Staphylococcus* spp. | N/A |
| 34 | *Pandoraea apista* | Not detected | N/A |
| 35 | *Granulicatella adiacens* | Not detected | N/A |
| 36 | *Bacillus* spp. | Not detected | N/A |
| 37 | *Staphylococcus haemolyticus,*  *Stenotrophomonas maltophilia* | *Staphylococcus* spp.,  *Stenotrophomonas maltophilia* | N/A |
| 38 | *Staphylococcus epidermidis* | *Staphylococcus* spp.  *Staphylococcus epidermidis* | *mecA/C* |
| 39 | *Enterococcus faecalis* | *Enterococcus faecalis* | Not detected |
| 40 | *Staphylococcus capitis* | *Staphylococcus* spp. | N/A |
| 41 | *Pseudomonas aeruginosa* | *Pseudomonas aeruginosa* | Not detected |
| 42 | *Stenotrophomonas maltophilia* | *Stenotrophomonas maltophilia* | N/A |
| 43 | *Staphylococcus epidermidis* | *Staphylococcus* spp.  *Staphylococcus epidermidis* | *mecA/C* |
| 44 | *Klebsiella pneumoniae* | *Enterobacterales*  *Klebsiella pneumoniae* group | Not detected |
| 45 | *Staphylococcus capitis,*  *Streptococcus mitis/oralis* | *Staphylococcus* spp.  *Streptococcus* spp. | N/A |
| 46 | *Staphylococcus cohnii* | *Staphylococcus* spp. | N/A |
| 47 | *Aeromonas veronii* complex | Not detected | N/A |
| 48 | *Staphylococcus capitis* | *Staphylococcus* spp. | N/A |
| 49 | *Staphylococcus hominis* | *Staphylococcus* spp. | N/A |
| 50 | *Staphylococcus epidermidis* | *Staphylococcus* spp.  *Staphylococcus epidermidis* | *mecA/C* |
| 51 | *Klebsiella pneumoniae* | *Enterobacterales*  *Klebsiella pneumoniae* group | Not detected |
| 52 | *Enterococcus faecium,*  *Corynebacterium striatum* | *Enterococcus faecium* | Not detected |
| 53 | *Staphylococcus epidermidis* | *Staphylococcus* spp.  *Staphylococcus epidermidis* | *mecA/C* |
| 54 | *Klebsiella pneumoniae* | *Enterobacterales*  *Klebsiella pneumoniae* group | CTX-M |
| 55 | *Streptococcus mitis/oralis* | *Streptococcus* spp. | N/A |
| 56 | *Staphylococcus epidermidis* | *Staphylococcus* spp.  *Staphylococcus epidermidis* | *mecA/C* |
| 57 | *Staphylococcus aureus* | *Staphylococcus* spp.  *Staphylococcus aureus* | Not detected |
| 58 | *Salmonella* group B | *Enterobacterales*  *Salmonella* spp. | Not detected |
| 59 | *Pseudomonas aeruginosa* | *Pseudomonas aeruginosa* | Not detected |
| 60 | *Streptococcus pyogenes* | *Streptococcus* spp.  *Streptococcus pyogenes* | N/A |
| 61 | *Brevibacterium luteolum* | Not detected | N/A |
| 62 | *Streptococcus mitis/oralis* | *Streptococcus* spp. | N/A |
| 63 | *Aeromonas veronii* complex | Not detected | N/A |
| 64 | *Corynebacterium striatum* | Not detected | N/A |
| 65 | *Rothia dentocariosa* | Not detected | N/A |
| 66 | *Staphylococcus epidermidis* | *Staphylococcus* spp.  *Staphylococcus epidermidis*,  Candida glabrata | *mecA/C* |
| 67 | *Escherichia coli* | *Enterobacterales*  *Escherichia coli* | Not detected |
| 68 | *Aeromonas veronii* complex | *Enterobacterales*  *Escherichia coli*,  *Pseudomonas aeruginosa,*  *Candida parapsilosis* | CTX-M, OXA-48-like |
| 69 | *Staphylococcus epidermidis* | *Staphylococcus* spp.  *Staphylococcus epidermidis* | *mecA/C* |
| 70 | *Staphylococcus capitis* | *Staphylococcus* spp. | N/A |
| 71 | *Candida tropicalis* | *Candida tropicalis* | N/A |
| 72 | *Roseomonas mucosa* | Not detected | N/A |
| 73 | *Staphylococcus epidermidis,*  *Escherichia coli,*  *Klebsiella pneumoniae* | *Staphylococcus* spp.  *Staphylococcus epidermidis*,  *Enterobacterales*  *Escherichia coli*,  *Klebsiella pneumoniae* group | CTX-M, *mecA/C* |
| 74 | *Staphylococcus epidermidis* | *Staphylococcus* spp.  *Staphylococcus epidermidis* | *mecA/C* |
| 75 | *Staphylococcus capitis* | *Staphylococcus* spp.  *Staphylococcus epidermidis* | *mecA/C* |
| 76 | *Staphylococcus epidermidis* | *Staphylococcus* spp.  *Staphylococcus epidermidis* | *mecA/C* |
| 77 | *Staphylococcus hominis* | *Staphylococcus* spp. | N/A |
| 78 | *Staphylococcus hominis* | *Staphylococcus* spp.  *Staphylococcus epidermidis* | *mecA/C* |
| 79 | *Staphylococcus capitis* | *Staphylococcus* spp. | N/A |
| 80 | *Klebsiella pneumoniae*,  *Pseudomonas aeruginosa* | *Enterobacterales*  *Klebsiella pneumoniae* group,  *Pseudomonas aeruginosa* | CTX-M |
| 81 | *Acinetobacter baumannii* complex | *Acinetobacter calcoaceticus-baumannii* complex | NDM |
| 82 | *Staphylococcus epidermidis,*  *Staphylococcus hominis* | *Staphylococcus* spp.  *Staphylococcus epidermidis* | *mecA/C* |
| 83 | *Staphylococcus epidermidis* | *Staphylococcus* spp.  *Staphylococcus epidermidis* | *mecA/C* |
| 84 | *Candida albicans* | *Candida albicans* | N/A |
| 85 | *Streptococcus mitis/oralis* | *Streptococcus* spp. | N/A |
| 86 | *Streptococcus peroris/infantis* | *Streptococcus* spp. | N/A |
| 87 | *Moraxella osloensis* | Not detected | N/A |
| 88 | *Staphylococcus epidermidis* | *Staphylococcus* spp.  *Staphylococcus epidermidis* | *mecA/C* |
| 89 | *Stenotrophomonas maltophilia* | *Stenotrophomonas maltophilia* | N/A |
| 90 | *Escherichia coli* | *Enterobacterales*  *Escherichia coli* | Not detected |
| 91 | *Staphylococcus epidermidis* | *Staphylococcus* spp.  *Staphylococcus epidermidis* | *mecA/C* |
| 92 | *Pseudomonas aeruginosa* | *Pseudomonas aeruginosa* | Not detected |
| 93 | *Salmonella* group D | *Enterobacterales*  *Salmonella* spp. | OXA-48-like |
| 94 | *Staphylococcus epidermidis* | *Staphylococcus* spp.  *Staphylococcus epidermidis* | *mecA/C* |
| 95 | *Escherichia coli* | *Enterobacterales*  *Escherichia coli* | Not detected |
| 96 | *Staphylococcus capitis* | *Staphylococcus* spp. | N/A |
| 97 | *Staphylococcus epidermidis* | *Staphylococcus* spp.  *Staphylococcus epidermidis* | *mecA/C* |
| 98 | *Staphylococcus haemolyticus,*  *Stenotrophomonas maltophilia* | *Staphylococcus* spp.,  *Stenotrophomonas maltophilia* | N/A |
| 99 | *Staphylococcus epidermidis* | *Staphylococcus* spp.  *Staphylococcus epidermidis* | *mecA/C* |
| 100 | *Stenotrophomonas maltophilia* | *Stenotrophomonas maltophilia* | N/A |
| 101 | *Stenotrophomonas maltophilia* | *Stenotrophomonas maltophilia* | N/A |
| 102 | *Staphylococcus epidermidis* | *Staphylococcus* spp.  *Staphylococcus epidermidis* | *mecA/C* |
| 103 | *Serratia marcescens* | *Enterobacterales*  *Serratia marcescens* | Not detected |
| 104 | *Staphylococcus epidermidis* | *Staphylococcus* spp.  *Staphylococcus epidermidis* | *mecA/C* |
| 105 | *Enterococcus faecalis* | *Enterococcus faecalis* | Not detected |
| 106 | *Stenotrophomonas maltophilia* | *Stenotrophomonas maltophilia* | N/A |
| 107 | *Staphylococcus hominis* | *Staphylococcus* spp. | N/A |
| 108 | *Salmonella* group D | *Enterobacterales*  *Salmonella* spp. | Not detected |
| 109 | *Pseudomonas mosselii* | Not detected | N/A |
| 110 | *Streptococcus dysgalactiae* | *Streptococcus* spp. | N/A |
| 111 | *Enterococcus faecalis,*  *Streptococcus mitis/oralis,*  *Streptococcus gordonii,*  *Rothia mucilaginosa* | *Enterococcus faecalis,*  *Staphylococcus* spp.,  *Streptococcus* spp. | Not detected |
| 112 | *Staphylococcus epidermidis* | *Staphylococcus* spp.  *Staphylococcus epidermidis* | *mecA/C* |
| 113 | *Pseudomonas aeruginosa* | *Pseudomonas aeruginosa* | Not detected |
| 114 | *Staphylococcus epidermidis* | *Staphylococcus* spp.  *Staphylococcus epidermidis* | *mecA/C* |
| 115 | *Bacillus* spp. | Not detected | N/A |
| 116 | *Brevundimonas diminuta/vesicularis,*  *Moraxella osloensis* | Not detected | N/A |
| 117 | *Staphylococcus aureus* | *Staphylococcus* spp.  *Staphylococcus aureus* | Not detected |
| 118 | *Brevundimonas diminuta/vesicularis,*  *Moraxella osloensis* | Not detected | N/A |
| 119 | *Staphylococcus epidermidis* | *Staphylococcus* spp.  *Staphylococcus epidermidis* | Not detected |
| 120 | *Staphylococcus haemolyticus* | *Staphylococcus* spp.  *Staphylococcus epidermidis* | *mecA/C* |
| 121 | *Klebsiella pneumoniae* | *Enterobacterales*  *Klebsiella pneumoniae* group | Not detected |
| 122 | *Staphylococcus epidermidis* | *Staphylococcus* spp.  *Staphylococcus epidermidis*,  *Candida parapsilosis* | *mecA/C* |
| 123 | *Acinetobacter baumannii* complex | *Acinetobacter calcoaceticus-baumannii* complex | NDM |
| 124 | *Candida tropicalis* | *Candida tropicalis* | N/A |
| 125 | *Micrococcus luteus* | Not detected | N/A |
| 126 | *Pseudomonas luteola* | Not detected | N/A |
| 127 | *Staphylococcus epidermidis* | *Staphylococcus* spp.  *Staphylococcus epidermidis* | *mecA/C* |
| 128 | *Acinetobacter baumannii* complex | *Acinetobacter calcoaceticus-baumannii* complex | NDM |
| 129 | *Klebsiella pneumoniae* | *Enterobacterales*  *Klebsiella pneumoniae* group | CTX-M |
| 130 | *Staphylococcus cohnii* | *Staphylococcus* spp. | N/A |
| 131 | *Stenotrophomonas maltophilia* | *Stenotrophomonas maltophilia* | N/A |
| 132 | *Streptococcus pyogenes* | *Streptococcus* spp.  *Streptococcus pyogenes* | N/A |
| 133 | *Escherichia coli* | *Enterobacterales*  *Escherichia coli* | CTX-M |
| 134 | *Staphylococcus epidermidis* | *Staphylococcus* spp.  *Staphylococcus epidermidis* | *mecA/C* |
| 135 | *Staphylococcus epidermidis* | *Staphylococcus* spp.  *Staphylococcus epidermidis* | *mecA/C* |
| 136 | *Staphylococcus aureus* | *Staphylococcus* spp.  *Staphylococcus aureus* | Not detected |
| 137 | *Pseudomonas aeruginosa* | *Pseudomonas aeruginosa* | Not detected |
| 138 | *Staphylococcus epidermidis* | *Staphylococcus* spp.  *Staphylococcus epidermidis* | *mecA/C* |
| 139 | *Enterococcus faecium* | *Enterococcus faecium* | *vanA/B* |
| 140 | *Salmonella* spp. | *Enterobacterales*  *Salmonella* spp. | Not detected |
| 141 | *Staphylococcus capitis* | *Staphylococcus* spp. | N/A |
| 142 | *Pseudomonas aeruginosa* | *Pseudomonas aeruginosa* | Not detected |
| 143 | *Staphylococcus hominis* | *Staphylococcus* spp.  *Staphylococcus epidermidis* | *mecA/C* |
| 144 | *Pantoea* spp. | *Enterobacterales* | Not detected |
| 145 | *Candida parapsilosis* complex | *Candida parapsilosis* | N/A |
| 146 | *Staphylococcus epidermidis,*  *Candida parapsilosis* complex | *Staphylococcus* spp.  *Staphylococcus epidermidis,*  *Candida parapsilosis* | *mecA/C* |
| 147 | *Burkholderia cenocepacia* | Not detected | N/A |
| 148 | *Streptococcus mitis/oralis* | *Streptococcus* spp. | N/A |
| 149 | *Burkholderia cenocepacia* | Not detected | N/A |
| 150 | *Staphylococcus cohnii* | *Staphylococcus* spp. | N/A |

N/A; Not applicable

**Supplementary S2**

**Performance of BCID2 in the detection of antimicrobial resistance genes compared with phenotypic antimicrobial susceptibility testing**

Sixty-one antimicrobial resistance genes were detected in 59 blood cultures, including mecA/C (n=44), blaCTX-M (n=8), blaNDM (n=6), blaOXA-48-like (n=2), and vanA/B (n=1). All mecA/C signals were associated with *Staphylococcus epidermidis* targets, including one polymicrobial culture containing *S. epidermidis* and *S. lugdunensis*. These 44 samples yielded positive cultures for *Staphylococcus* spp., and 37 were concordant with conventional culture growth of methicillin-resistant *S. epidermidis*, including three polymicrobial cultures in which methicillin-resistant *S. epidermidis* and methicillin-resistant *S. hominis* were identified in the same specimen. The remaining seven cultures yielded non-epidermidis staphylococci on conventional culture, all of which were methicillin resistant.

Four blaCTX-M-harboring *Klebsiella pneumoniae* and three *Escherichia coli* isolates were detected byBCID2, corresponding to phenotypic resistance to cefuroxime, ceftriaxone, and cefepime, while remaining susceptible to carbapenems. All blaCTX-M-positive organisms exhibited susceptible, intermediate, or resistant phenotypes to amoxicillin/clavulanate and piperacillin/tazobactam. The remaining blaCTX-M, together with a blaOXA-48-like gene, was detected in a blood culture with false-positive BCID2 identification of *C. parapsilosis*, *E. coli* and *P. aeruginosa*, whereas conventional culture identified *Aeromonas veronii* complex as the true causative organism (Supplementary Table S1, No.68).

Six phenotypically carbapenem-resistant *Acinetobacter baumannii* isolates were identified, all of which were correctly predicted by the BCID2 via blaNDM detection. Discrepancies occurred in one blaOXA-48-like-positive Salmonella isolate and one vanA/B-positive *Enterococcus faecium* isolate identified by BCID2; however, conventional culture showed susceptibility to carbapenems and vancomycin, respectively. These findings were confirmed by minimal inhibitory concentration method using ETEST® strips (bioMérieux, Marcy-l'Étoile, France). No organisms carrying blaIMP, blaKPC, blaVIM, or mcr-1 were detected by BCID2, and none were found on subsequent antimicrobial susceptibility testing.
